# Probing Polymorphic Stacking Domains in Mechanically Exfoliated Two-Dimensional Nanosheets Using Atomic Force Microscopy and Ultralow-Frequency Raman Spectroscopy

**DOI:** 10.3390/nano14040339

**Published:** 2024-02-09

**Authors:** Chengjie Pei, Jindong Zhang, Hai Li

**Affiliations:** Key Laboratory of Flexible Electronics (KLOFE), Institute of Advanced Materials (IAM), Nanjing Tech University (NanjingTech), 30 South Puzhu Road, Nanjing 211816, China

**Keywords:** transition metal dichalcogenides, stacking order, ultralow-frequency Raman spectroscopy, electrostatic screening, atomic force microscopy

## Abstract

As one of the key features of two-dimensional (2D) layered materials, stacking order has been found to play an important role in modulating the interlayer interactions of 2D materials, potentially affecting their electronic and other properties as a consequence. In this work, ultralow-frequency (ULF) Raman spectroscopy, electrostatic force microscopy (EFM), and high-resolution atomic force microscopy (HR-AFM) were used to systematically study the effect of stacking order on the interlayer interactions as well as electrostatic screening of few-layer polymorphic molybdenum disulfide (MoS_2_) and molybdenum diselenide (MoSe_2_) nanosheets. The stacking order difference was first confirmed by measuring the ULF Raman spectrum of the nanosheets with polymorphic stacking domains. The atomic lattice arrangement revealed using HR-AFM also clearly showed a stacking order difference. In addition, EFM phase imaging clearly presented the distribution of the stacking domains in the mechanically exfoliated nanosheets, which could have arisen from electrostatic screening. The results indicate that EFM in combination with ULF Raman spectroscopy could be a simple, fast, and high-resolution method for probing the distribution of polymorphic stacking domains in 2D transition metal dichalcogenide materials. Our work might be promising for correlating the interlayer interactions of TMDC nanosheets with stacking order, a topic of great interest with regard to modulating their optoelectronic properties.

## 1. Introduction

In recent years, two-dimensional (2D) layered materials have attracted great interest because of their extensive applications in electrocatalysis, optoelectronics, energy storage, sensing, and other fields [1,2,3,4,5]. Among the available 2D materials, the arrangement of adjacent layers, i.e., the stacking order between nanosheets, dominates the polytypes of 2D materials. It is known that the optoelectronic and electrocatalytic properties of 2D materials are highly sensitive to the stacking order [6,7], also termed the crystal phase, along with the size, thickness, and composition [8,9]. For instance, the ABA (Bernal) and ABC (rhombohedral) stacking orders have been shown to have a significant influence on the electronic and optical properties of graphene [10,11,12]. MoS_2_ with a hexagonal phase (2H-MoS_2_) is the most thermodynamically stable structure and is useful for low-cost photoelectronic applications, while MoS_2_ with a trigonal phase (1T-MoS_2_) has shown promising performance as an electrocatalyst [13,14]. It has been reported that MoS_2_ with a rhombohedral phase (3R-MoS_2_) is an excellent material for nonlinear optical devices [15]. In addition, robust sliding ferroelectricity has also been demonstrated in a 3R-MoS_2_-based dual-gate FET device [16].

To date, several techniques have been used to distinguish the stacking order or twisted structure in monolayer and few-layer 2D materials, including Raman spectroscopy [17,18,19,20,21,22], transmission electron microscopy (TEM) [23,24,25,26], scanning transmission electron microscopy (STEM) [27,28,29], second-harmonic generation (SHG) [15,30,31,32,33,34], third-harmonic generation (THG) [35,36,37], infrared scanning nearfield optical microscopy (IR-SNOM) [38,39,40,41], absorption spectral mapping [42], phase imaging via tapping-mode atomic force microscopy (AFM) [43], scanning microwave impedance microscopy [44], PL mapping [45], and Kelvin probe force microscopy (KPFM) [46,47,48]. Due to the complex atomic arrangement in transition metal dichalcogenides (TMDCs), the impact of the stacking order on the properties of TMDCs is more intriguing than that on graphene [49]. Although ultralow-frequency (ULF) Raman spectroscopy has been extensively used to identify the crystal phases or stacking orders of few-layer TMDCs [50,51], the spatial resolution of Raman mapping is unsatisfactory. Shear-anisotropy-driven microscopy has successfully been employed to image the crystalline orientation of MoS_2_ nanosheets, but it is dependent on the flexibility of monolayer and bilayer MoS_2_ nanosheets [52]. Since the presence of rich polymorphs or stacking orders in TMDCs endows them with diverse electric, catalytic, optical, and magnetic properties [15,27,53,54,55,56,57], it is essential to correlate the interlayer interactions with the stacking order in depth. Therefore, it is highly desirable to develop a simple, rapid, and high-resolution method with which to directly image the stacking order distribution in 2D TMDC materials.

In this work, the polymorphic stacking domains in mechanically exfoliated few-layer molybdenum disulfide (MoS_2_) and molybdenum diselenide (MoSe_2_) nanosheets were directly revealed using electrostatic force microscopy (EFM), high-resolution atomic force microscopy (HR-AFM), and ULF Raman spectroscopy. The distribution of non-2H (including 3R and other types) and 2H stacking orders in the few-layer MoS_2_ and MoSe_2_ nanosheets was imaged using EFM with a high resolution. The various stacking orders were then confirmed using HR-AFM and ULF Raman spectroscopy. Our results indicate that EFM is a promising and effective method with which to directly identify the stacking order distribution in few-layer TMDCs nanosheets with a mixed crystal phase.

## 2. Experimental Section

### 2.1. Sample Preparation

A SiO_2_/Si silicon wafer with an oxide layer thickness of 300 nm was cut into 1.2 cm × 1.2 cm square pieces and washed via ultrasonication in a mixture of acetone and deionized water (with a volume ratio of 1:1) for 20 min. Then, the SiO_2_/Si pieces were heated at 110 °C for 30 min in a mixture of concentrated sulfuric acid and hydrogen peroxide (with a volume ratio of 1:1). After that, the SiO_2_/Si pieces were washed with deionized water three times and then dried using purified nitrogen.

Monolayer and few-layer MoSe_2_ and MoS_2_ nanosheets were deposited on freshly cleaned 300 nm SiO_2_/Si substrates using mechanically exfoliating 2H-phase MoS_2_ and MoSe_2_ crystals (HQ Graphene, Groningen, The Netherlands).

### 2.2. Sample Characterization

Optical images were captured using an optical microscope (Axio Scope A1, Zeiss, Jena, Germany). The thickness of the 2D nanosheets on the 300 nm SiO_2_/Si substrates was analyzed using ULF Raman spectroscopy, AFM, and an optical contrast method. The Image J software (https://imagej.net) was used to analyze the contrast difference between the MoS_2_ nanosheets and the 300 nm SiO_2_/Si substrate by splitting the grayscale optical image [58]. As shown in Figure S1 in the Supporting Information (SI), the red and green channel images could be split from the color channel image. Then, the optical contrast value differences between the MoS_2_ nanosheets and the SiO_2_/Si substrate in the red and green channels were obtained by measuring the optical contrast values using Image J. The optical contrast value difference of 1–10L MoS_2_ nanosheets is plotted in Figure S2 in the SI as a standard chart for thickness identification. Furthermore, the optical contrast value differences of the MoS_2_ nanosheets with an unknown thickness were compared with the standard chart, which could be used to distinguish the layer numbers in the MoS_2_ nanosheets.

High-frequency and ultralow-frequency Raman and photoluminescence spectra were obtained using a Raman microscope (LabRAM HR Evolution, Horiba, Kyoto, Japan). A laser of 532 nm was focused through a 100× objective lens at room temperature. A laser power of 8.2 μW and a 60 s accumulation time were chosen to avoid damaging the sample. The Raman band of a Si substrate at 520 cm^−1^ was used as a reference for calibration. An atomic force microscope (Multimode 8, Bruker, Billerica, MA, USA) was used to obtain the thickness and atomic lattice structures of MoS_2_ and MoSe_2_ nanosheets. EFM results were collected using the SCM-PIT tip. The cantilever frequency was 75 kHz, and the spring constant was 3 N/m. The scan rate of the EFM test was 1 Hz. The tip bias was applied from −2 V to +2 V, and the lift height was changed from 20 nm to 150 nm.

## 3. Results and Discussion

**Scheme 1:**
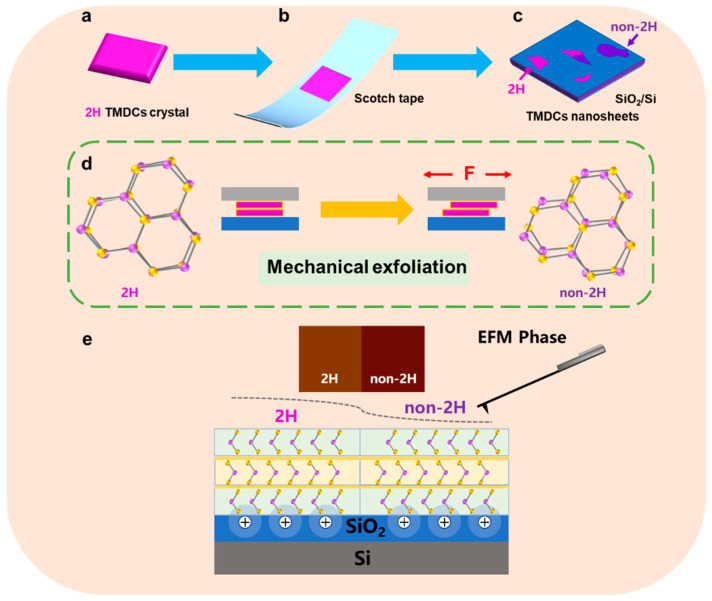
Scheme depicting the preparation and characterization of mechanically exfoliated MoS_2_ and MoSe_2_ with different stacking orders. (**a**,**b**) Peeling off of the multilayer nanosheets from the 2H-phase crystal using scotch tape. (**c**,**d**) Deposition of the 2H- and non-2H-phase MoS_2_ or MoSe_2_ nanosheets from the bulk 2H crystal via mechanical exfoliation. (**e**) EFM characterization of 2H- and non-2H-phase MoS_2_ or MoSe_2_ nanosheets.

The experimental process is shown in Scheme 1. The few-layer MoS_2_ and MoSe_2_ nanosheets were exfoliated from the 2H-phase crystals to the 300 nm SiO_2_/Si substrate (Scheme 1a–c). Firstly, the 2H-phase crystal (Scheme 1a) was peeled off using scotch tape (Scheme 1b). After multilayer MoS_2_ and MoSe_2_ nanosheets were deposited on scotch tape and brought into contact with the SiO_2_/Si substrate, an external force was usually applied to the scotch tape to rub the multilayer nanosheets. Due to the van der Waals interaction between the multilayer nanosheets and the SiO_2_/Si substrate, the monolayer to few-layer nanosheets could remain on the substrate after the scotch tape was removed (Scheme 1c). In this process, the stacking order of the nanosheets could be altered from a 2H phase to a non-2H phase due to the shear force applied between the scotch tape and the SiO_2_/Si substrate (Scheme 1d). Then, AFM was used to measure the surface topography of the nanosheets, and EFM was employed to characterize their stacking domain distribution (Scheme 1e).

**Figure 1:**
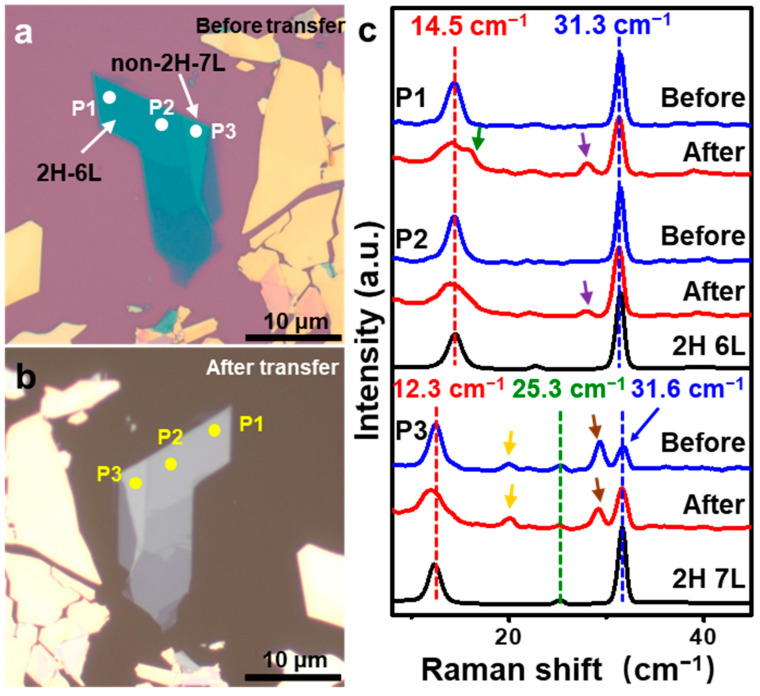
Optical images of the MoS_2_ nanosheets consisting of 2H 6L and non-2H 7L layers (**a**) before and (**b**) after their transfer onto a PDMS film. (**c**) The ULF Raman spectra of regions P1, P2, and P3 in the 6L and 7L MoS_2_ layers before and after the transfer. The red dashed lines are referred to the breathing-mode peak, the blue and green dashed lines are referred to the shear-mode peaks.

To investigate the origin of the stacking order alternation in the TMDC nanosheets, the few-layer MoS_2_ nanosheet was first mechanically exfoliated on the SiO_2_/Si substrate and then transferred onto a flexible polydimethylsiloxane (PDMS) film. Afterward, a Raman microscope was used to monitor the evolution of the ULF and high-frequency Raman peaks of the MoS_2_ nanosheets before and after the transfer. As shown in Figure 1a, the MoS_2_ nanosheet consisting of 6L and 7L layers was deposited on a 300 nm SiO_2_/Si substrate via mechanical exfoliation. The thickness of the MoS_2_ layer was confirmed by measuring the optical contrast and ULF Raman spectra [58,59]. The optical contrast difference values in the red and green channels of the upper-left MoS_2_ nanosheet were −118 and 25, respectively, which could be assigned to the 6L MoS_2_ from Figure S2 in the SI. In addition, these values for the upper-right MoS_2_ nanosheet were −107 and 30 and could be assigned to the 7L MoS_2_.

Three regions (referred to as P1, P2, and P3 in Figure 1a) on the MoS_2_ nanosheet were selected to present the change in stacking order by monitoring their ULF Raman spectra before and after being transferred onto the PDMS film (Figure 1b). Regions P1 and P2 were located on the 6L nanosheet, while region P3 was located on the 7L MoS_2_ nanosheet. Regions P1 and P2 showed ULF shear- and breathing-mode peaks located at 31.3 and 14.5 cm^−1^, respectively, which were consistent with those of the 6L MoS_2_ with a 2H phase (Figure 1c), indicating that they maintained the 2H phase after being deposited on the 300 nm SiO_2_/Si substrate. After being transferred onto the PDMS film, regions P1 and P2 showed two new shear-mode peaks at 28.3 (indicated by the purple arrows in Figure 1c) and 16.1 cm^−1^ (indicated by the green arrow in Figure 1c), indicating the formation of a non-2H-phase stacking order. As shown in Figure 1c, the 2H-phase 7L MoS_2_ showed three characteristic ULF Raman peaks located at 12.3, 25.3, and 31.6 cm^−1^, respectively. Region P3 showed additional ULF Raman peaks located at 20.1 (indicated by the yellow arrows in Figure 1c) and 29.3 cm^−1^ (indicated by the brown arrows in Figure 1c) in addition to the characteristic Raman peaks, implying a stacking order change to a non-2H phase after mechanical exfoliation. Moreover, the intensity ratio of the Raman peaks at 29.3 cm^−1^ to 31.6 cm^−1^ changed from 1.38 to 0.54 after the nanosheets were transferred onto the PDMS film, indicating that the stacking order was further changed during the transfer process.

During the mechanical exfoliation process, it is possible to cause a stacking order change due to the shear-force-assisted movement of the constituent layers, thereby forming nanosheets with polymorphic domains. Such a phenomenon has also been reported in relation to the stacking order change in mechanically exfoliated few-layer graphene and 2H-TaS_2_ [22,60]. The aforementioned phenomena confirmed the stacking order change in the MoS_2_ nanosheets during the mechanical exfoliation and transfer processes.

**Figure 2:**
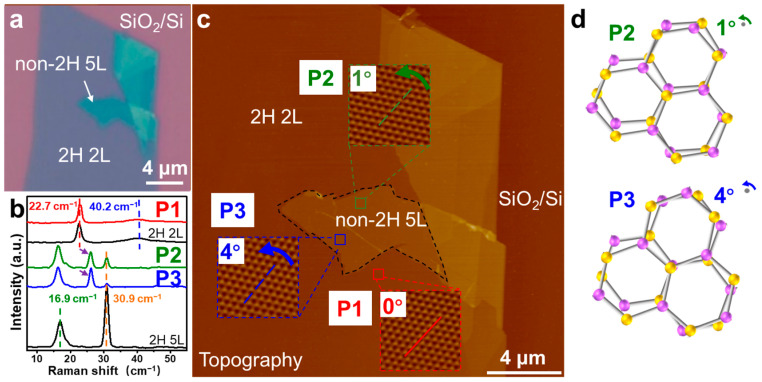
(**a**) Optical and (**c**) AFM topography images. (**b**) ULF Raman spectra of the MoS_2_ nanosheet on the 300 nm SiO_2_/Si substrate. The red and blue dashed lines are referred to the shear- and breathing-mode peaks of 2H 2L MoS_2_, the orange and green dashed lines are referred to the shear- and breathing-mode peaks of 2H 5L MoS_2_. The purple arrows are referred to the new shear-mode peaks of non-2H 5L MoS_2_. (**d**) Simulated atomic lattice images of regions P2 and P3 shown in (**c**) and according to the measured twist angle.

The stacking order change in the few-layer MoS_2_ nanosheet was further revealed via HR-AFM and ULF Raman spectroscopy, yielding detailed results. As shown in Figure 2a, the MoS_2_ nanosheet consisting of 2L and 5L layers was deposited on a 300 nm SiO_2_/Si substrate via mechanical exfoliation. The thickness of the MoS_2_ nanosheet was confirmed through AFM as well as optical contrast measurements. The stacking order was revealed through ULF Raman spectroscopy. As shown in Figure 2b, the 2L MoS_2_ layer showed a 2H phase since it had a ULF shear-mode peak at 22.7 cm^−1^ and a breathing-mode peak at 40.2 cm^−1^. The 2H-phase 5L MoS_2_ had characteristic ULF Raman shear- and breathing-mode peaks located at 30.9 and 16.9 cm^−1^, respectively. Furthermore, besides these characteristic ULF Raman peaks, the 5L MoS_2_ layer shown in Figure 2a exhibited an additional ULF shear-mode peak at 26.0 cm^−1^ (indicated by the purple arrows in Figure 2b), indicating that it had a non-2H phase. The non-2H 5L MoS_2_ layer could have been formed through the stacking of another 3L MoS_2_ layer on top of the 2H-phase 2L MoS_2_ layer, as indicated by the AFM height image shown in Figure 2c. Since the bottom 2L MoS_2_ layer has a 2H-phase and the as-stacked 5L MoS_2_ layer has a non-2H phase, the top 3L MoS_2_ layer should be stacked in a non-2H manner.

In order to reveal the stacking order of the non-2H 5L MoS_2_ layer, HR-AFM was performed to image the atomic lattice orientation of the bottom 2H-phase 2L MoS_2_ layer and the top non-2H 3L MoS_2_ layer, referred to as regions P1, P2, and P3 in Figure 2c. Although regions P2 and P3 were located in the same nanosheet, they showed different ULF Raman peaks (Figure 2c). The ULF Raman peak located at 30.9 cm^−1^ of region P2 showed a higher intensity than that of region P3, implying that the two might have different stacking orders. Compared to region P1, regions P2 and P3 showed anti-clockwise twist angles of 1° and 4° (as shown in the insets in Figure 2c,d), respectively. As shown in Figure 2c, there was a wrinkle between regions P2 and P3, indicating that the origin of the stacking order differed between them. A similar phenomenon was also observed in the non-2H 4L MoS_2_ consisting of a 1L MoS_2_ layer stacked on top of a 2H-phase 3L MoS_2_ layer with a twist angle of 3° (Figure S3 in SI).

Besides the non-2H stacked 3L MoS_2_, there was another 1L MoS_2_ layer stacked on top of the 2H-phase 2L MoS_2_, forming a 3L MoS_2_ layer, as shown in Figure S4 in the SI. Although the atomic lattice orientation of the 3L MoS_2_ layers (regions P2 and P3, shown in Figure S4c) was the same as that of the bottom 2H-phase 2L MoS_2_ layer (region P1, shown in Figure S3c), the ULF Raman spectra indicate that the crystal phases of the 3L MoS_2_ layers in regions P2 and P3 were different (Figure S4b). As shown in Figure S4b, the 3L MoS_2_ in region P3 showed mixed shear- and breathing-mode peaks around 28.1 cm^−1^, which are consistent with those of the 2H-phase 3L MoS_2_ (indicated by the bottom black curve in Figure S4b), confirming its 2H phase, while the 3L MoS_2_ in region P2 showed a ULF shear-mode peak located at 16.2 cm^−1^ and a breathing-mode peak (purple arrow in Figure S3b) located at 27.6 cm^−1^, respectively, indicating its 3R phase [51]. Since the 2L MoS_2_ in region P1 was in a 2H phase, the 3R phase of the 3L MoS_2_ in region P2 could be attributed to the sliding of the top 1L MoS_2_ layer (Figure S4d).

**Figure 3:**
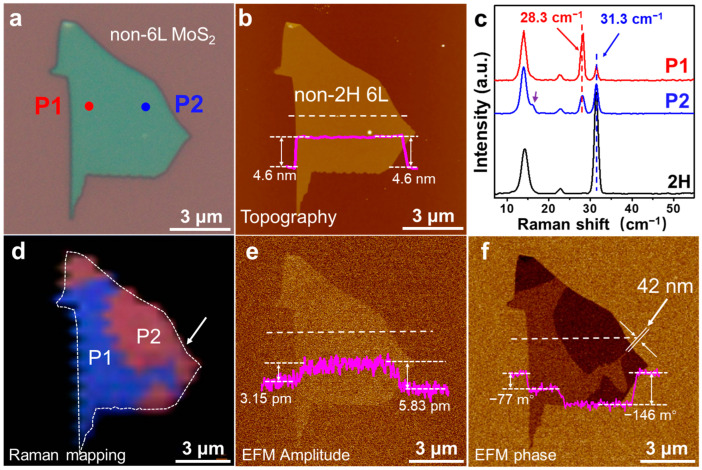
(**a**) Optical image, (**b**) AFM topography image, (**c**) ULF Raman spectra, the red and blue dashed lines are referred to the shear-mode peaks of non-2H 6L MoS_2_, (**d**) Raman mapping in the range of 25.5 to 35.5 cm^−1^, (**e**) EFM amplitude image, and (**f**) EFM phase image of the 6L MoS_2_ sample with different stacking orders.

Figure 3a,b show the optical and AFM topography images of a mechanically exfoliated MoS_2_ nanosheet on a 300 nm SiO_2_/Si substrate. The height of the MoS_2_ nanosheet is 4.6 nm, indicating that its thickness is 6L. Compared to the 2H-phase 6L MoS_2_ nanosheet (indicated by the black curve in Figure 3c), the ULF Raman spectra of regions P1 and P2 show new shear-mode peaks at 28.3 cm^−1^. In addition, the intensity of the shear-mode peak at 31.3 cm^−1^ is considerably lower than that of the 2H-phase 6L MoS_2_. Therefore, the 6L MoS_2_ nanosheet shown in Figure 3a,b should be in a non-2H phase. Although regions P1 and P2 show similar ULF Raman peaks (indicated by the blue and red curves in Figure 3c), they show different intensity ratios of the peak at 28.3 cm^−1^ to the peak at 31.3 cm^−1^, which were 3.38 and 0.58, respectively. Furthermore, a small shear-mode peak (indicated by the purple arrow) at 16.1 cm^−1^ appeared in the ULF Raman spectra of region P2. These results indicate that the stacking orders in regions P1 and P2 are different, which was also confirmed by the ULF Raman mapping of regions P1 and P2 in the range of 25.5 to 35.5 cm^−1^ (Figure 3d).

It is known that there are charged impurities trapped between mechanically exfoliated 2D nanosheets and SiO_2_/Si substrates under ambient conditions [61,62,63]. Such charged impurities induce an electric field that can be screened by 2D nanosheets [64,65,66,67]. Moreover, the interlayer coupling between MoS_2_ layers plays a crucial role in the electrostatic screening of an electric field [63], different from that of graphene. To reveal the electrostatic screening by MoS_2_ nanosheets with various stacking orders, EFM was employed. As shown in Figure 3e, there is quite a faint difference between regions P1 and P2 in the EFM amplitude image. Region P1 shows an amplitude difference of 3.15 pm compared to the SiO_2_/Si substrate and that of region P2 shows a slight increase to 5.83 pm, which could be attributed to the low sensitivity of amplitude detection in the EFM test. The cantilever could be considered an oscillator with some damping, while the interaction between the probe and sample would affect the amplitude. However, a certain period of time was required for adjusting the oscillation amplitude, which led to almost no difference in the amplitude profile between regions P1 and P2 (Figure 3e). However, phase detection in the EFM test was instant and sensitive, meaning that the phase shift difference was more obvious than the amplitude change. Regions P1 and P2 show obvious contrast differences (−77 m° and −146 m°) in the EFM phase image (Figure 3f), implying different forms of electrostatic screening induced by the stacking order.

**Figure 4:**
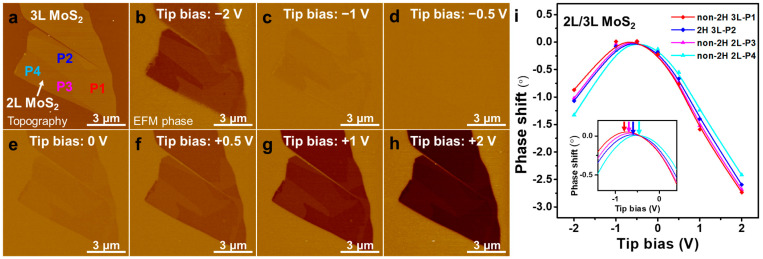
(**a**) AFM topography image. (**b**–**h**) EFM phase images of 2L and 3L MoS_2_ nanosheets shown in (**a**), with the tip bias changed from −2 V to +2 V. (**i**) EFM phase shift as a function of the applied tip bias.

To further investigate the influence of stacking order on electrostatic screening, a scanning bias voltage (−2 V to +2 V) was applied to the EFM tip to generate an external electric field with different strengths [68]. Firstly, a MoS_2_ nanosheet consisting of 2L and 3L layers with different stacking orders was deposited on the 300 nm SiO_2_/Si substrate and imaged using AFM (Figure 4a). As shown in Figure 4b–h, with the change in tip bias voltage, the contrast of different stacking domains (referred to as regions P1, P2, P3, and P4 in Figure 4a) in the EFM phase images was obviously changed, indicating the noticeable electrostatic screening ability of the stacking order. The phase shift results are summarized in Figure 4i. In our EFM test, the resonant frequency shift (∆∅) was larger than the cantilever frequency. Thus, the EFM phase shift is expressed as follows [65]:

(1) $$∆\phi =\frac{\partial F}{\partial z}\cdot \frac{Q}{k}$$

Here, *F* is the driving force, i.e., electrostatic force in the EFM test; *z* is the motion displacement of the AFM tip; *k* is the spring constant of the cantilever; and *Q* is the quality factor of the cantilever. If the capacitive interaction between the AFM tip and the sample is considered to be ideal capacitance, the relationship between electrostatic force and applied voltage can be described as follows [63]:

(2) $$F=\frac{1}{2}\frac{\partial C}{\partial z}{\left(V_{tip}-V_{s}\right)}^{2}$$

Here, *C* is the capacitance between the AFM tip and the sample; *V_tip_* is the DC bias voltage; and *V_s_* is the effective surface potential of the sample. By combining Equations (1) and (2), the relationship between the force gradient (*∂F*/*∂z*) and bias voltage can be written as

(3) $$\frac{\partial F}{\partial z}=\frac{1}{2}\frac{\partial^{2}C}{\partial z^{2}}{\left(V_{tip}-V_{s}\right)}^{2}$$

As a result, the phase shift values of regions P1, P2, P3, and P4 in Figure 4a show an obvious parabolic dependence on the voltage applied to the AFM tip, which is similar to that of MoS_2_ nanosheets with different thicknesses [69]. Moreover, the apex of each curve indicates that the applied voltage on the tip is equal to the *V_s_*. The curve of 2H-phase 3L MoS_2_ reaches its apex at an applied voltage of −0.59 V, indicating the *V_s_* of 2H-phase 3L MoS_2_, while the *V_s_* of non-2H 3L MoS_2_ is −0.71 V. In addition, the *V_s_* of non-2H-phase 2L MoS_2_ in region P3 is −0.68 V, while that of non-2H-phase 2L MoS_2_ in region P4 is −0.44 V. Therefore, the surface potential of MoS_2_ nanosheets with different stacking orders could also be detected via EFM phase imaging. For the MoS_2_ nanosheets with the same thicknesses, the apex of the parabola is sensitive to the stacking order, indicating the latter’s significant effect on electrostatic screening. The EFM phase shifts of the MoS_2_ nanosheets with different stacking orders are highly dependent on the lift height during the scanning process. EFM phase measurement on a 15L MoS_2_ nanosheet was also performed, with the lift height changed from 20 to 150 nm (Figure S5 in SI). The phase shift was about 0.94° when the lift height was set to 20 nm. It decreased rapidly to 0.08° when the lift height increased to 150 nm. By plotting the EFM phase shift as a function of the lift height, it can be observed that the phase shift of the MoS_2_ nanosheets with different stacking domains decreased exponentially as the lift height increased (Figure S5 in SI), a result which aligns with the standard EFM response [70,71,72,73].

**Figure 5:**
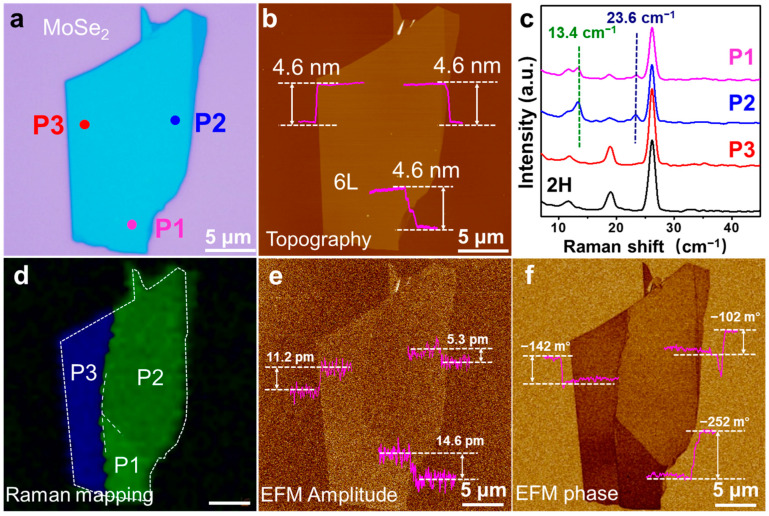
(**a**) Optical image, (**b**) AFM topography image, (**c**) ULF Raman spectra, the green and blue dashed lines are referred to the shear-mode peaks of non-2H 6L MoSe_2_, (**d**) Raman mapping in the range of 12.4 to 14.4 cm^−1^ and 17.1 to 20.7 cm^−1^, (**e**) EFM amplitude image, and (**f**) EFM phase image of the 6L MoSe_2_ sample with different stacking orders.

EFM phase imaging is also effective in distinguishing the stacking orders of other TMDC nanosheets. As shown in Figure 5a, a MoSe_2_ nanosheet with a uniform thickness was deposited on a 300 nm SiO_2_/Si substrate. The thickness of the MoSe_2_ nanosheet was 4.6 nm, indicating that it was 6L thick. Three regions (referred to as P1, P2, and P3 in Figure 5a) on the MoSe_2_ nanosheet were selected to reveal the stacking order domains using their ULF Raman spectra and EFM phase imaging. Region P3 in Figure 5a has three characteristic ULF Raman peaks at 11.9, 19.0, and 26.2 cm^−1^, which are consistent with those of the 2H-6L MoSe_2_ nanosheet (indicated by the black curve in Figure 5c), indicating that it was in the 2H phase, while regions P1 and P2 in Figure 5a also show additional Raman peaks at 13.4 and 23.6 cm^−1^, respectively, demonstrating that they were in a non-2H phase. In addition, regions P1 and P2 in Figure 5a show the same Raman peaks but different intensities at 11.9 and 13.4 cm^−1^, implying that they also have slightly different stacking orders. The Raman mapping result shown in Figure 5d clearly presents the different contrast between regions P2 and P3, while the contrast difference between regions P1 and P2 is almost indistinguishable.

Regions P1, P2, and P3 in Figure 5a show EFM amplitude shifts of 14.6, 5.3, and 11.2 pm, respectively, compared to the SiO_2_/Si substrate, for which the shifts are difficult to distinguish. Compared to the EFM amplitude image, the EFM phase image clearly shows the contrast differences of regions P1 and P2 with different stacking orders (Figure 5e,f). Regions P1, P2, and P3 show EFM phase shifts of −252, −102, and −142 m° compared to the SiO_2_/Si substrate, respectively. These results prove that EFM phase imaging is a direct and powerful method for visualizing the stacking order distributions of TMDCs on a 300 nm SiO_2_/Si substrate.

Similarly, bias voltage (−2 V to +2 V) was also applied on the same MoSe_2_ nanosheet to investigate the electrostatic screening of stacking orders (Figure S6 in SI). As shown in Figure S6, when the tip bias voltage changed from −2 V to +2 V, the EFM phase shifts of different stacking domains (regions P1, P2, and P3) also changed correspondingly, indicating the influence of stacking order on electrostatic screening ability. As shown in Figure S6i, the phase shift values of regions P1, P2, and P3 had a quadratic relation with the applied voltage of the AFM tip, which was similar to that of the MoS_2_ nanosheets shown in Figure 4. For the MoSe_2_ nanosheets with the same thicknesses, the apex of the parabola was also sensitive to the stacking order. These results show that the stacking order had a greater impact on the electrostatic screening effect than layer thickness.

## 4. Conclusions

In summary, we investigated few-layer MoS_2_ and MoSe_2_ nanosheets with various stacking orders using ULF Raman spectroscopy, HR-AFM, and EFM. The correlation between stacking order and interlayer interaction as well as the electrostatic screening of 2D materials were explored in detail. In the TMDC nanosheets with different stacking orders, the electrostatic screening contrast was consistent with the stacking domain distribution. Through EFM characterization, phase mapping with higher contrast and resolution could be obtained. Therefore, EFM phase imaging was proposed to be a direct and accurate method for characterizing polymorphic domain distribution in mechanically exfoliated 2D materials. Compared to thickness, the stacking order had a more significant impact on electrostatic screening. Our work could be helpful for studying the influence of stacking order on the optical and electronic properties of polymorphic TMDC samples.

## Data Availability

Data are contained within the article and Supplementary Materials.

## Supplementary Materials

The following supporting information can be downloaded at: https://www.mdpi.com/article/10.3390/nano14040339/s1, Figures S1 and S2: Optical contrast images of MoS_2_ nanosheets; Figure S3 and S4: ULF Raman spectra and high-resolution AFM images of polymorphic MoS_2_; Figure S5: EFM images of polymorphic MoS_2_; Figure S6: EFM images of polymorphic MoSe_2_.
