# Supplementary material for: Probing Polymorphic Stacking Domains in Mechanically Exfoliated Two-Dimensional Nanosheets Using Atomic Force Microscopy and Ultralow-Frequency Raman Spectroscopy
Source: Nanomaterials (Basel). 2024 Feb 9;14(4):339. doi: 10.3390/nano14040339 (PMC10892501; doi:10.3390/nano14040339)
Supplement: Supplementary file 1 [file nanomaterials-14-00339-s001.zip › nanomaterials-2812734-supplementary.pdf]

Supporting Information

Probing Polymorphic Stacking Domains in Mechanically Exfoliated Two-Dimensional Nanosheets Using Atomic Force Microscopy and Ultralow-Frequency Raman Spectroscopy

Chengjie Pei, Jindong Zhang and Hai Li *

Key Laboratory of Flexible Electronics (KLOFE), Institute of Advanced Materials (IAM), Nanjing Tech University (NanjingTech), 30 South Puzhu Road, Nanjing 211816, China; iampcj@njtech.edu.cn (C.P.); iamjdzhang@njtech.edu.cn (J.Z.)
* Correspondence: iamhli@njtech.edu.cn

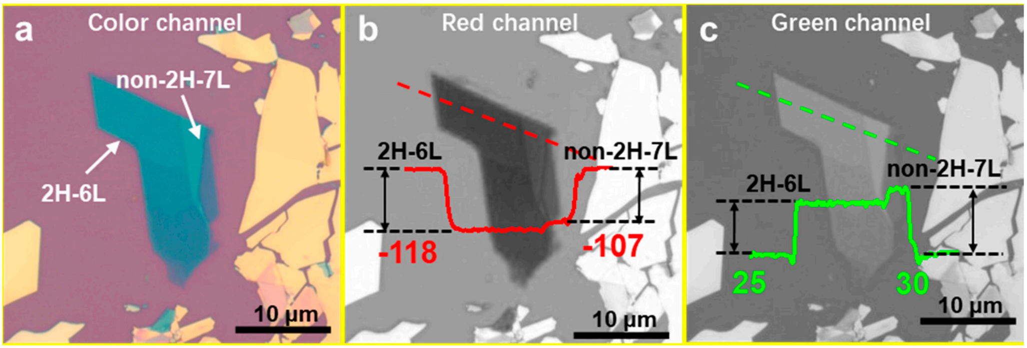

Figure S1. (a) color channel image of mechanical exfoliated MoS_2. (b-c) red and green channel images of mechanical exfoliated MoS_2.

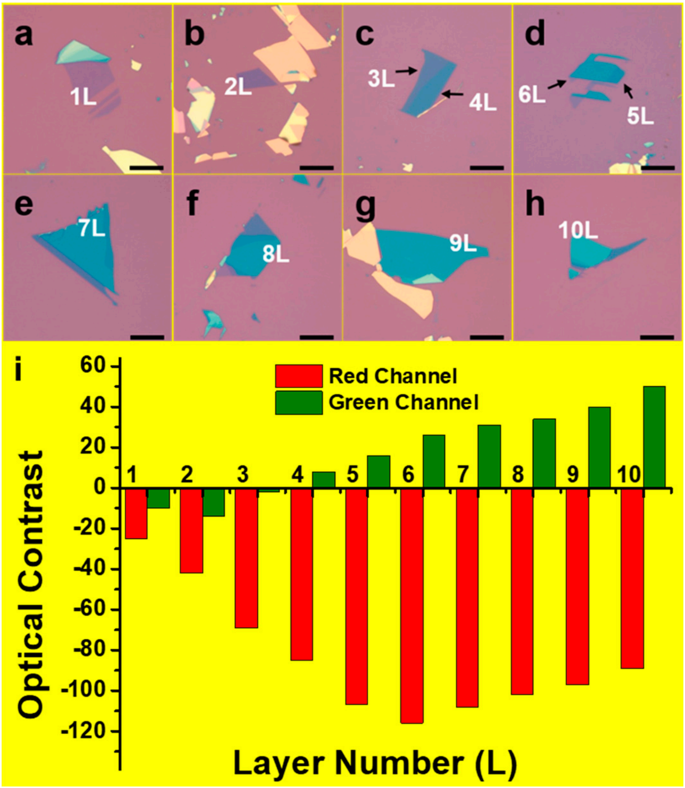

Figure S2. (a-h) Optical images of mechanical exfoliated 1-10L MoS_2, respectively. All the scale bars are 10 μm. (i) Plot of optical contrast difference of 1-10L MoS_2 under red and green channels.

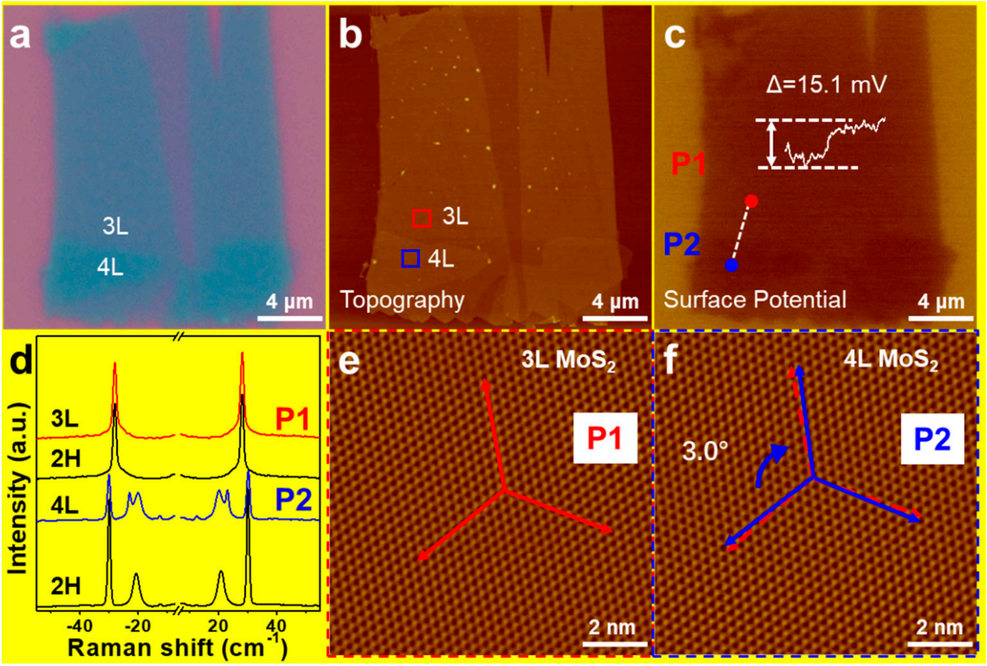

Figure S3. (a) Optical image, (b) topography image, (c) surface potential image, (d) Raman spectra, (e-f) and atomic lattice images of polymorphic MoS_2 nanosheet.

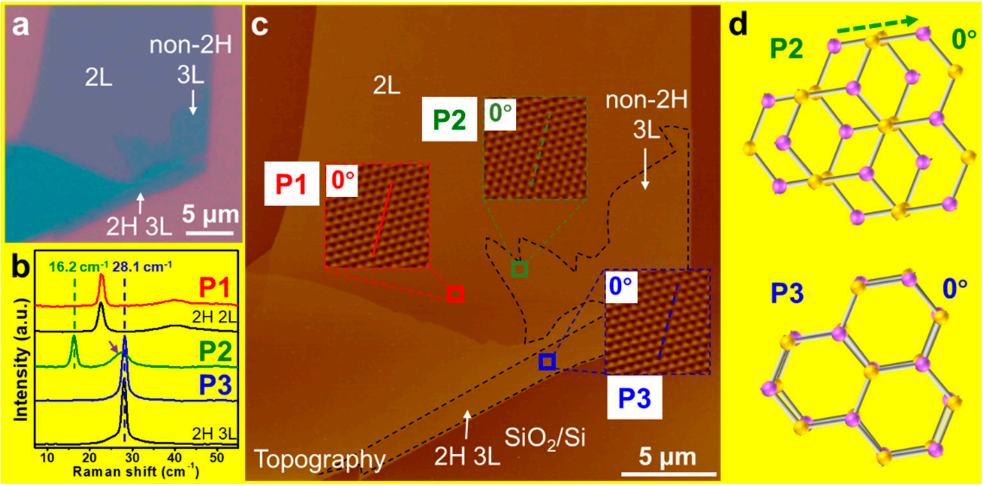

Figure S4. (a) Optical and (c) AFM topography images, (b) ULF Raman spectra of MoS_2 nanosheet on 300 nm SiO_2/Si substrate. (d) Simulated atomic lattice images of regions P2 and P3 shown in (c) and according to the measured twist angle.

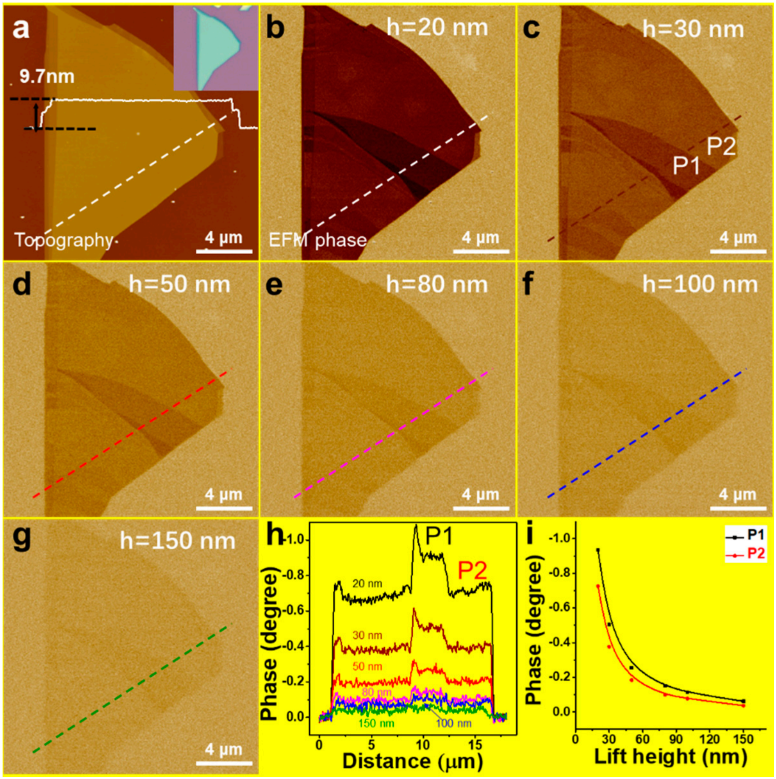

Figure S5. (a) Topography image and (b-g) EFM phase images of polymorphic MoS_2 nanosheet at various lift heights. (h) The phase of dashed lines and the phase shift (i) vs lift height obtained in the EFM measurement on polymorphic MoS_2 nanosheet.

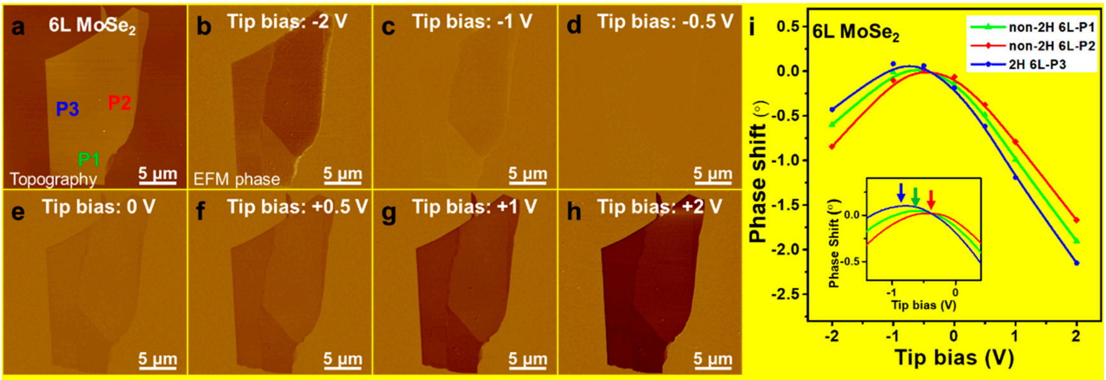

Figure S6. (a) AFM topography image, (b-h) EFM phase images of 6L $MoSe_2$ nanosheets shown in (a) with the tip bias changed from -2 V to +2 V. (i) EFM phase shift as a function of the applied tip bias.
